# Supplementary material for: High prevalence of heteroresistance in Staphylococcus aureus is caused by a multitude of mutations in core genes
Source: PLoS Biol. 2024 Jan 4;22(1):e3002457. doi: 10.1371/journal.pbio.3002457 (PMC10766187; doi:10.1371/journal.pbio.3002457)
Supplement: S1 Fig — MICs were measured with a single E-test. The dotted vertical lines represent the threshold for clinical resistance according to EUCAST. (A) MICs of DAP (daptomycin), (B) MICs of GEN (gentamicin), (C) MICs of LNZ (linezolid), (D) MICs of OXA (oxacillin), (E) MICs of TEC (teicoplanin), and (F) MICs of VAN (vancomycin). (PDF) [file pbio.3002457.s001.pdf]

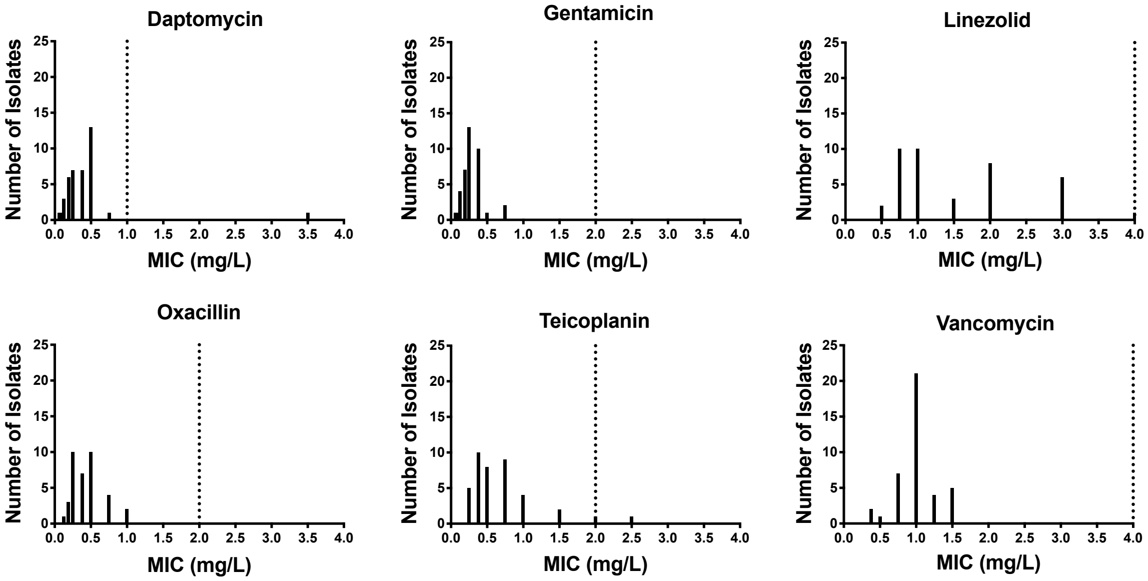

S1 Fig. Frequency and distribution of MIC values of six different antibiotics for 40 clinical *S. aureus* isolates. MICs were measured with a single E-test. The dotted vertical lines represent the threshold for clinical resistance according to EUCAST. A: MICs of DAP (daptomycin), B: MICs of GEN (gentamicin), C. MICs of LNZ (linezolid); D. MICs of OXA (oxacillin), E: MICs of TEC (teicoplanin); and F: MICs of VAN (vancomycin).
