# Supplementary figures and images for: High prevalence of heteroresistance in Staphylococcus aureus is caused by a multitude of mutations in core genes
Source: PLoS Biol. 2024 Jan 4;22(1):e3002457. doi: 10.1371/journal.pbio.3002457 (PMC10766187; doi:10.1371/journal.pbio.3002457)

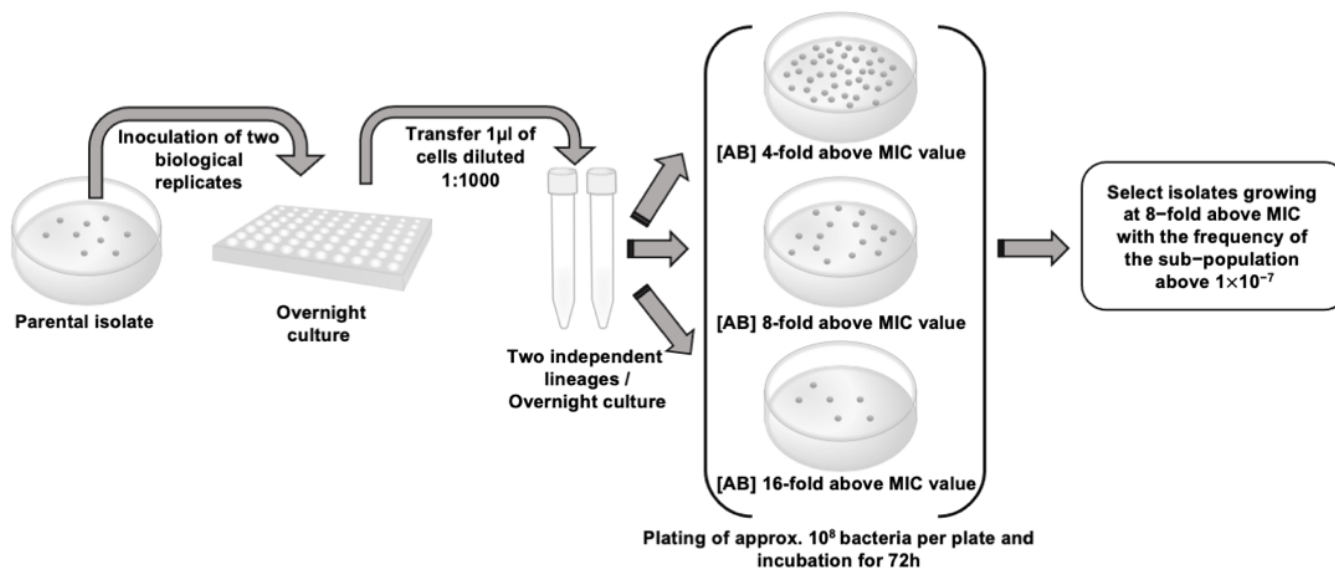

**Fig S2. Schematic representation of the pre-screening procedure.**

Supplement: S2 Fig — (PDF) [file pbio.3002457.s002.pdf]
