# Supplementary material for: High prevalence of heteroresistance in Staphylococcus aureus is caused by a multitude of mutations in core genes
Source: PLoS Biol. 2024 Jan 4;22(1):e3002457. doi: 10.1371/journal.pbio.3002457 (PMC10766187; doi:10.1371/journal.pbio.3002457)
Supplement: S3 Fig — Isolates showing growth of subpopulations at 8- or 16-fold the MIC value were selected for PAP tests to confirm an HR phenotype. The total number of susceptible isolates used for prescreening of each antibiotic was: DAP (daptomycin): 39, GEN (gentamicin): 39, LNZ (linezolid): 40, OXA (oxacillin): 37, TEC (teicoplanin): 39, and VAN (vancomycin): 40. (PDF) [file pbio.3002457.s003.pdf]

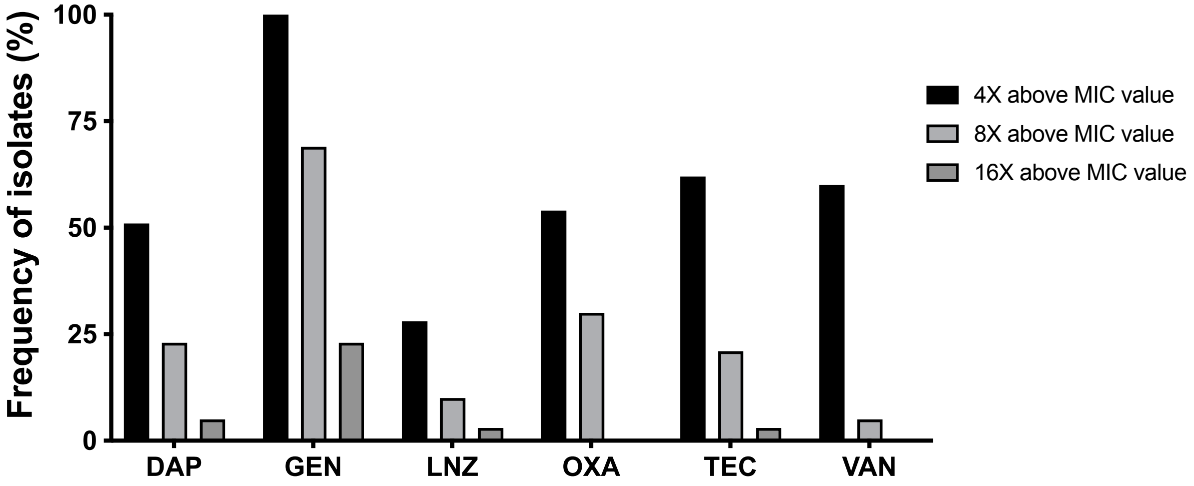

S3 Fig. Frequency of susceptible *S. aureus* isolates growing at antibiotic concentrations (4X, 8X and 16X) above MIC determined in the pre-screen for each antibiotic. Isolates showing growth of subpopulations at 8- or 16-fold the MIC value were selected for PAP tests to confirm a HR phenotype. The total number of susceptible isolates used for pre-screening of each antibiotic was: DAP (daptomycin): 39, GEN (gentamicin): 39, LNZ (linezolid): 40, OXA (oxacillin): 37, TEC (teicoplanin): 39, and VAN (vancomycin): 40.
