# Supplementary material for: High prevalence of heteroresistance in Staphylococcus aureus is caused by a multitude of mutations in core genes
Source: PLoS Biol. 2024 Jan 4;22(1):e3002457. doi: 10.1371/journal.pbio.3002457 (PMC10766187; doi:10.1371/journal.pbio.3002457)
Supplement: S4 Fig — Each curve is based on the average of 3 independent experiments. The red line on the graph marks the non-HR isolates. The horizontal dotted lines indicate the subpopulation frequency cutoff (1 × 10^−7^) used to identify HR isolates. For raw data, see S2 Table. (PDF) [file pbio.3002457.s004.pdf]

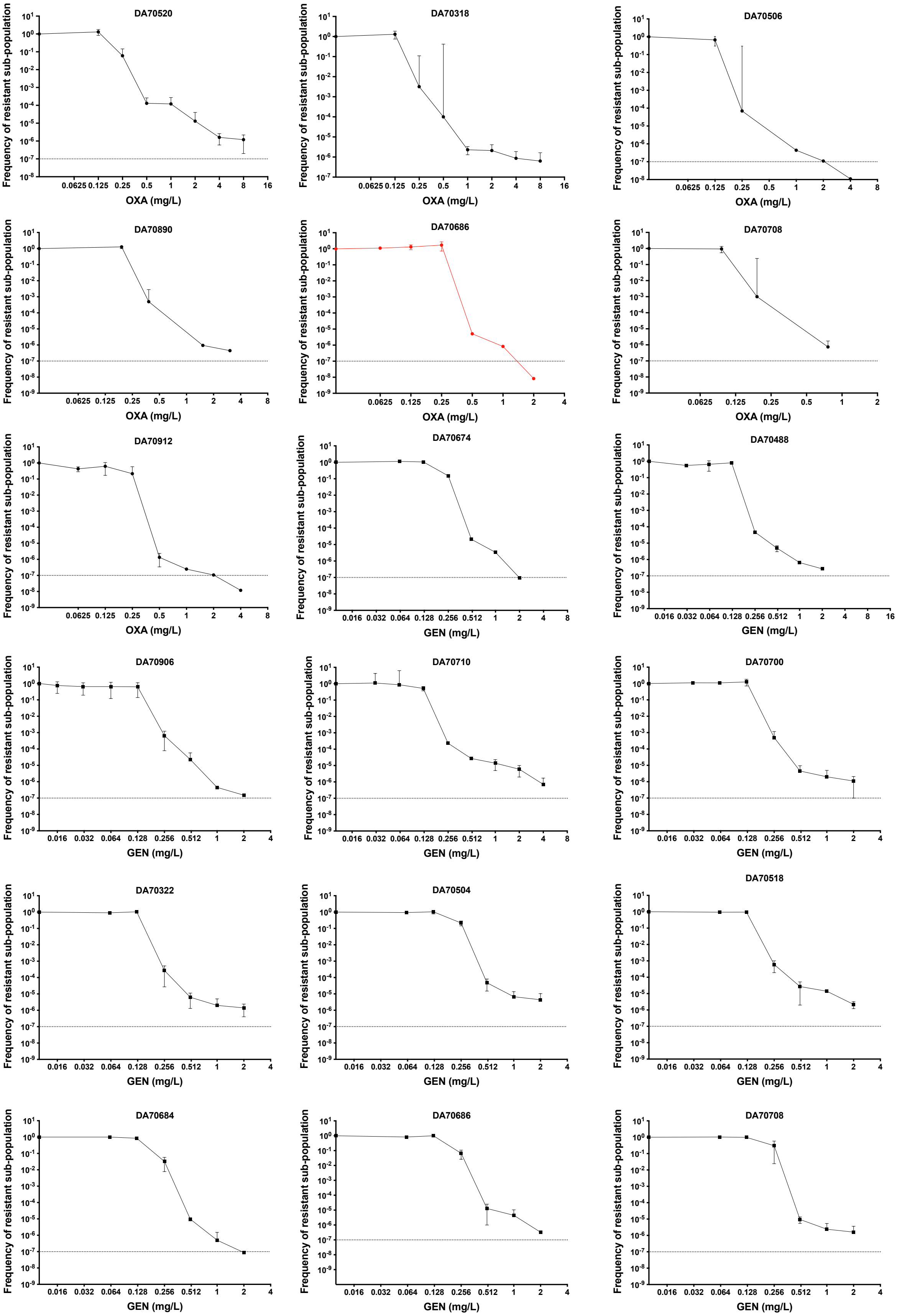

DA70520
Frequency of resistant sub-population
OXA (mg/L)
DA70318
Frequency of resistant sub-population
OXA (mg/L)
DA70506
Frequency of resistant sub-population
OXA (mg/L)
DA70890
Frequency of resistant sub-population
OXA (mg/L)
DA70686
Frequency of resistant sub-population
OXA (mg/L)
DA70708
Frequency of resistant sub-population
OXA (mg/L)
DA70912
Frequency of resistant sub-population
OXA (mg/L)
DA70674
Frequency of resistant sub-population
GEN (mg/L)
DA70488
Frequency of resistant sub-population
GEN (mg/L)
DA70906
Frequency of resistant sub-population
GEN (mg/L)
DA70710
Frequency of resistant sub-population
GEN (mg/L)
DA70700
Frequency of resistant sub-population
GEN (mg/L)
DA70322
Frequency of resistant sub-population
GEN (mg/L)
DA70504
Frequency of resistant sub-population
GEN (mg/L)
DA70518
Frequency of resistant sub-population
GEN (mg/L)
DA70684
Frequency of resistant sub-population
GEN (mg/L)
DA70686
Frequency of resistant sub-population
GEN (mg/L)
DA70708
Frequency of resistant sub-population
GEN (mg/L)

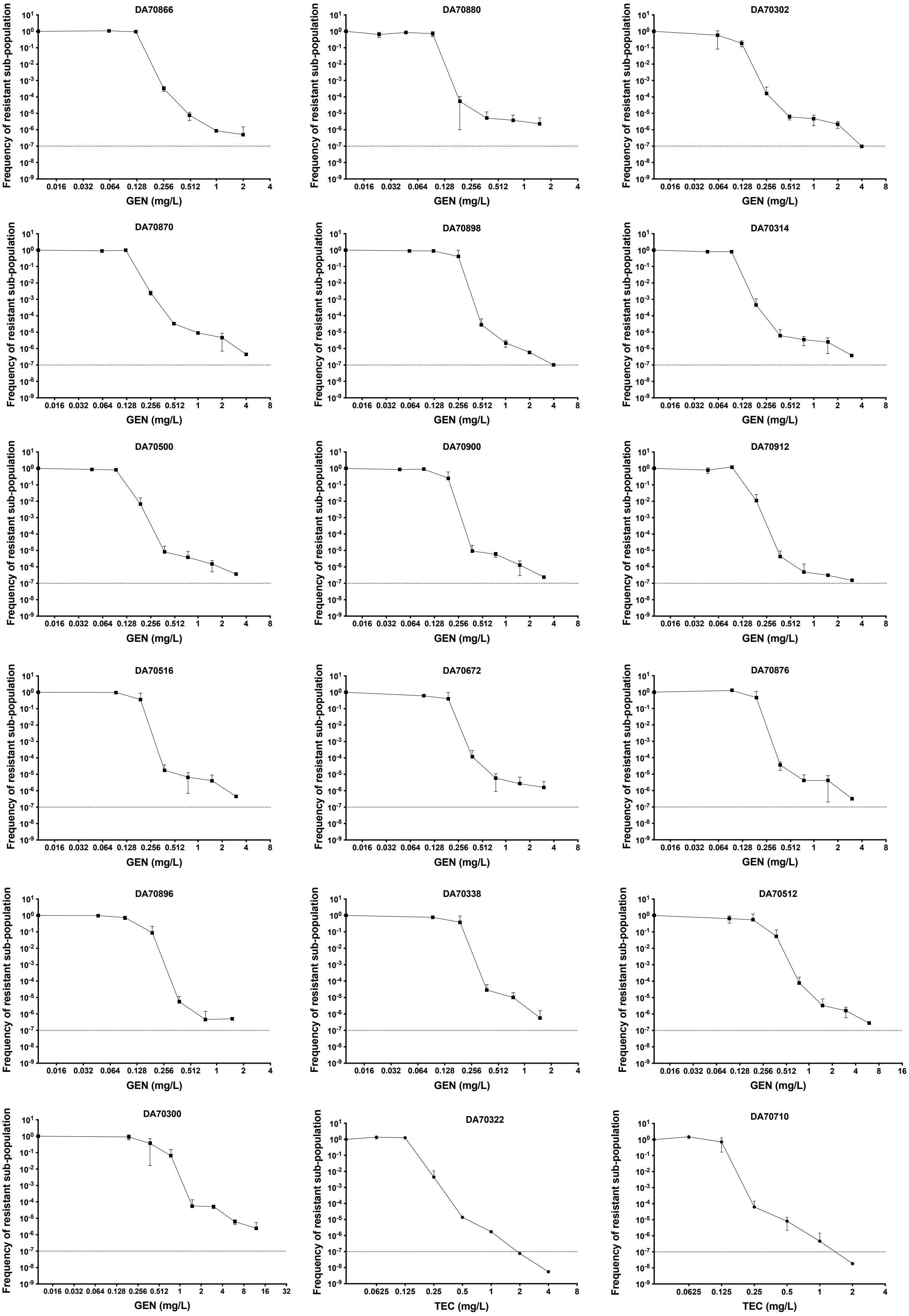
DA70866
DA70880
DA70302
DA70870
DA70898
DA70314
DA70500
DA70900
DA70912
DA70516
DA70672
DA70876
DA70896
DA70338
DA70512
DA70300
DA70322
DA70710
Frequency of resistant sub-population
GEN (mg/L)
TEC (mg/L)

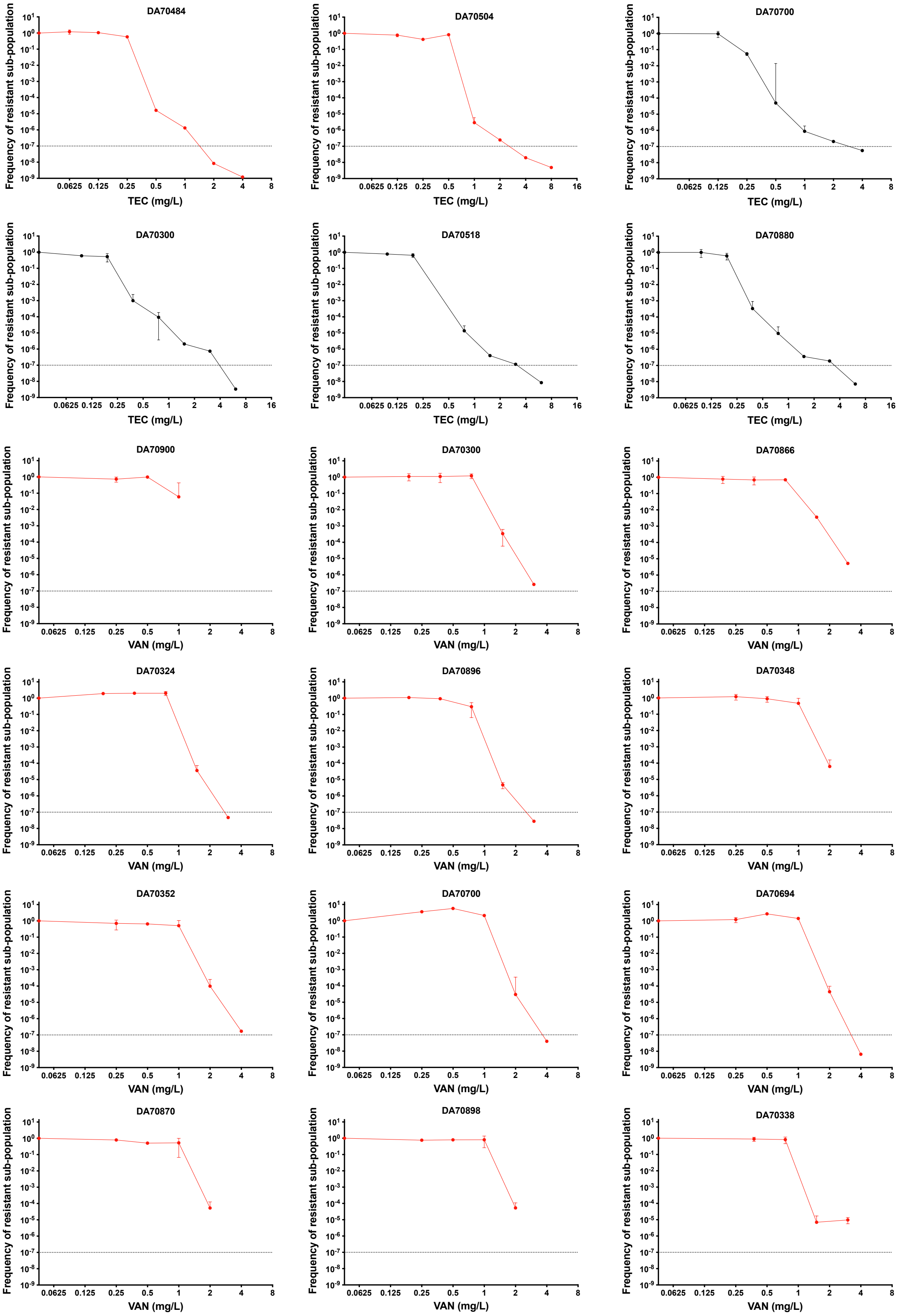
DA70484
DA70504
DA70700
DA70300
DA70518
DA70880
DA70900
DA70300
DA70866
DA70324
DA70896
DA70348
DA70352
DA70700
DA70694
DA70870
DA70898
DA70338
Frequency of resistant sub-population
TEC (mg/L)
VAN (mg/L)
0.0625
0.125
0.25
0.5
1
2
4
8
16

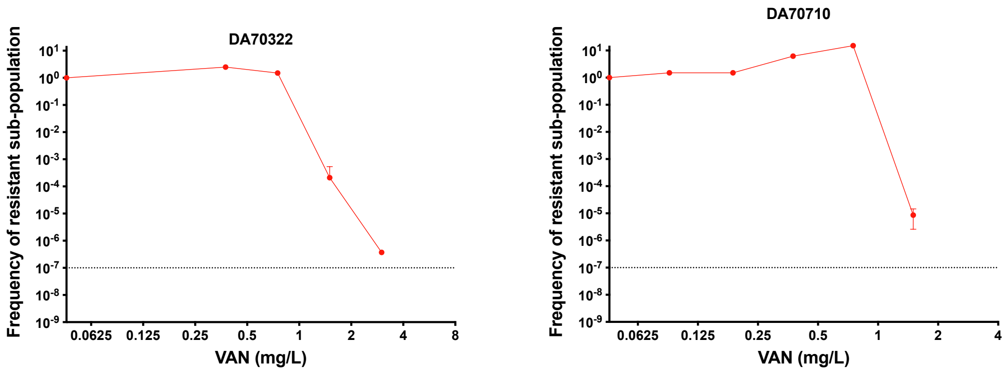

S4 Fig. Population analysis profile (PAP) of all isolates identified as probable HR in the pre-screen. Each curve is based on the average of three independent experiments. The red line on the graph marks the non-HR isolates. The horizontal dotted lines indicate the subpopulation frequency cut-off ($1x10^{-7}$) used to identify HR isolates. See Table S1 for CFU data for each antibiotic.
