# Supplementary material for: High prevalence of heteroresistance in Staphylococcus aureus is caused by a multitude of mutations in core genes
Source: PLoS Biol. 2024 Jan 4;22(1):e3002457. doi: 10.1371/journal.pbio.3002457 (PMC10766187; doi:10.1371/journal.pbio.3002457)
Supplement: S5 Fig — Symbols ** and **** indicate high significance in the Mann–Whitney test, with the p-values of <0.0001 and 0.0073, respectively, while “ns” shows nonsignificant differences. (A) DAP (daptomycin), (B) GEN (gentamicin), (C) OXA (oxacillin), and (D) TEC (teicoplanin). (PDF) [file pbio.3002457.s005.pdf]

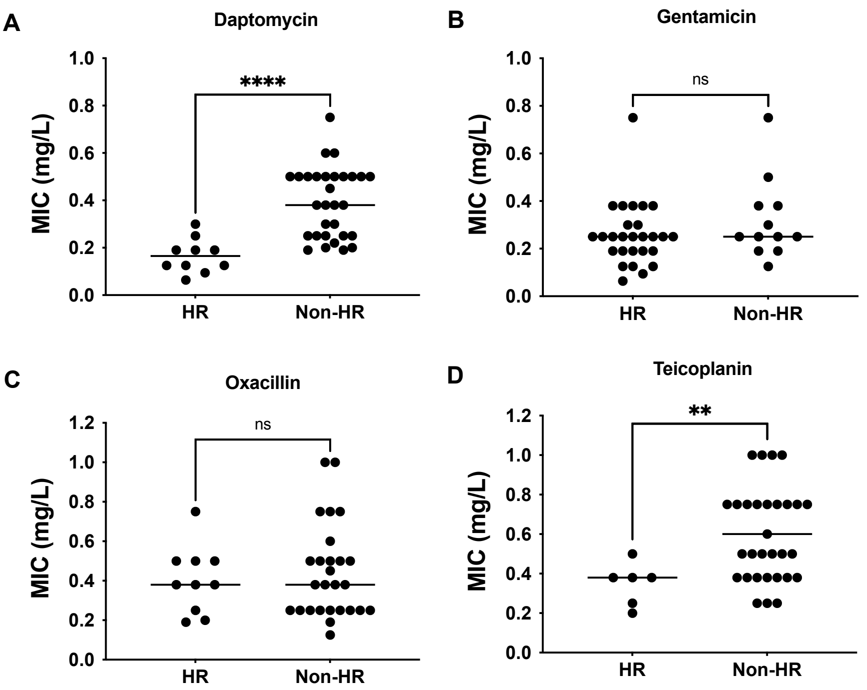

S5 Fig. Distribution of HR and non-HR isolates of *S. aureus* as a function of MIC value. Symbols ** and **** indicate high significance in the Mann-Whitney test, with the p-values of < 0.0001 and 0.0073, respectively, while "ns" shows non-significant differences. A. DAP (daptomycin), B. GEN (gentamicin), C. OXA (oxacillin), and D. TEC (teicoplanin).
