# Supplementary material for: High prevalence of heteroresistance in Staphylococcus aureus is caused by a multitude of mutations in core genes
Source: PLoS Biol. 2024 Jan 4;22(1):e3002457. doi: 10.1371/journal.pbio.3002457 (PMC10766187; doi:10.1371/journal.pbio.3002457)
Supplement: S6 Fig — DA numbers for each parental strain and mutant are on the x-axis. The relative growth rates (normalized to the growth rate of the parental strain) and MIC values are represented with bars and dots, respectively. Parental isolates (dark bars and white dots) are always followed by the mutants (gray bars and black dots) isolated from that specific parental isolate. Relative growth rates are based on 5 biological replicates and the error bars indicate standard deviation. MICs are based on a single E-test. (A) DAP (daptomycin), (B) GEN (gentamicin), (C) OXA (oxacillin), and (D) TEC (teicoplanin). (PDF) [file pbio.3002457.s006.pdf]

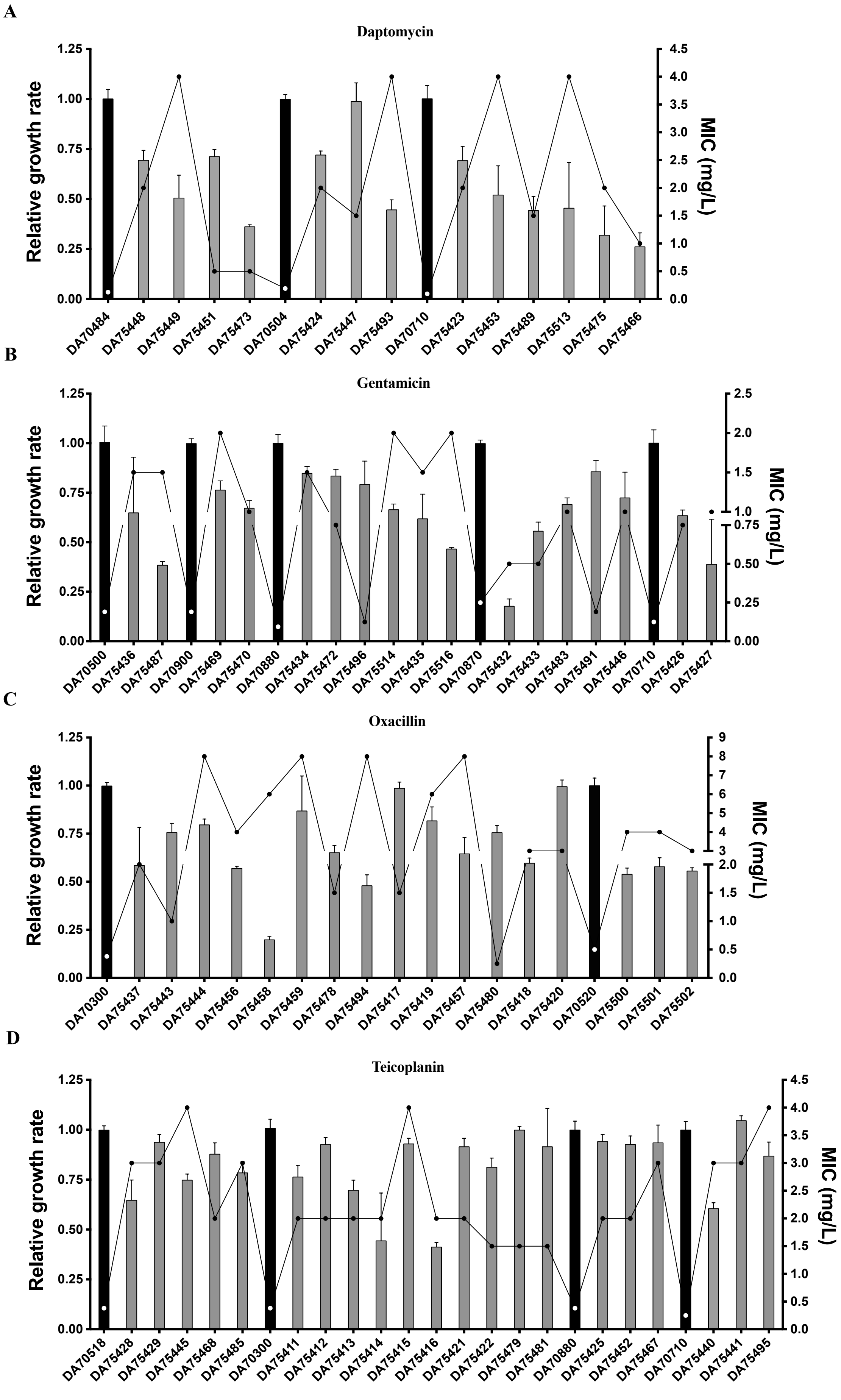
A
Daptomycin
Relative growth rate
MIC (mg/L)
DA70484
DA75448
DA75449
DA75451
DA75473
DA70504
DA75424
DA75447
DA75493
DA70710
DA75423
DA75453
DA75489
DA75513
DA75475
DA75466
B
Gentamicin
Relative growth rate
MIC (mg/L)
DA70500
DA75436
DA75487
DA70900
DA75469
DA75470
DA70880
DA75434
DA75472
DA75496
DA75514
DA75435
DA75516
DA70870
DA75432
DA75433
DA75483
DA75491
DA75446
DA70710
DA75426
DA75427
C
Oxacillin
Relative growth rate
MIC (mg/L)
DA70300
DA75437
DA75443
DA75444
DA75456
DA75458
DA75459
DA75478
DA75494
DA75417
DA75419
DA75457
DA75480
DA75418
DA75420
DA70520
DA75500
DA75501
DA75502
D
Teicoplanin
Relative growth rate
MIC (mg/L)
DA70518
DA75428
DA75429
DA75445
DA75468
DA75485
DA70300
DA75411
DA75412
DA75413
DA75414
DA75415
DA75416
DA75421
DA75422
DA75479
DA75481
DA70880
DA75425
DA75452
DA75467
DA70710
DA75440
DA75441
DA75495

S6 Fig. **Relative growth rate and resistance profile for parental strains and all resistant mutants belonging to each parental strain**. DA numbers for each parental strain and mutant are on the x-axis. The relative growth rates (normalized to the growth rate of the parental strain) and MIC values are represented with bars and dots, respectively. Parental isolates (dark bars and white dots) are always followed by the mutants (grey bars and black dots) isolated from that specific parental isolate. Relative growth rates are based on five biological replicates and the error bars indicate standard deviation. MICs are based on a single E-test. A. DAP (daptomycin), B. GEN (gentamicin), C. OXA (oxacillin), and D. TEC (teicoplanin).
