# Supplementary material for: High prevalence of heteroresistance in Staphylococcus aureus is caused by a multitude of mutations in core genes
Source: PLoS Biol. 2024 Jan 4;22(1):e3002457. doi: 10.1371/journal.pbio.3002457 (PMC10766187; doi:10.1371/journal.pbio.3002457)
Supplement: S7 Fig — Mutants and parental isolates are indicated with black and red dots, respectively. (A) DAP (daptomycin), (B) GEN (gentamicin), (C) OXA (oxacillin), and (D) TEC (teicoplanin). (PDF) [file pbio.3002457.s007.pdf]

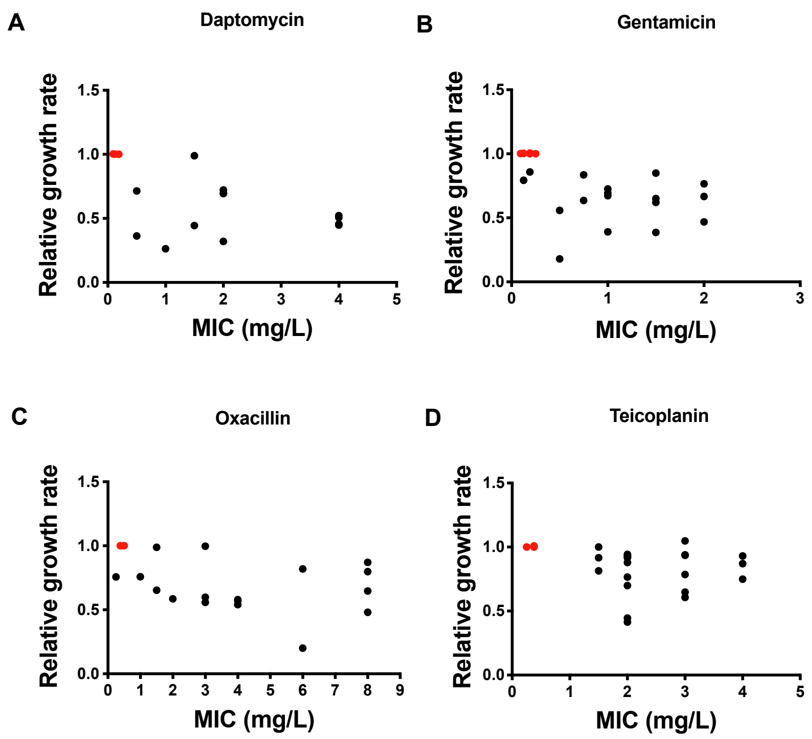

S7 Fig. **Correlations between relative growth rate and resistance level**. Mutants and parental isolates are indicated with black and red dots, respectively. A. DAP (daptomycin), B. GEN (gentamicin), C. OXA (oxacillin), and D. TEC (teicoplanin). Data are from Fig. S6.
