# Supplementary material for: High prevalence of heteroresistance in Staphylococcus aureus is caused by a multitude of mutations in core genes
Source: PLoS Biol. 2024 Jan 4;22(1):e3002457. doi: 10.1371/journal.pbio.3002457 (PMC10766187; doi:10.1371/journal.pbio.3002457)
Supplement: S9 Fig — Mutants (DA number below) belong to parental isolates indicated on top of the graph. Fs indicates frame shift and * stop-codon. (PDF) [file pbio.3002457.s009.pdf]

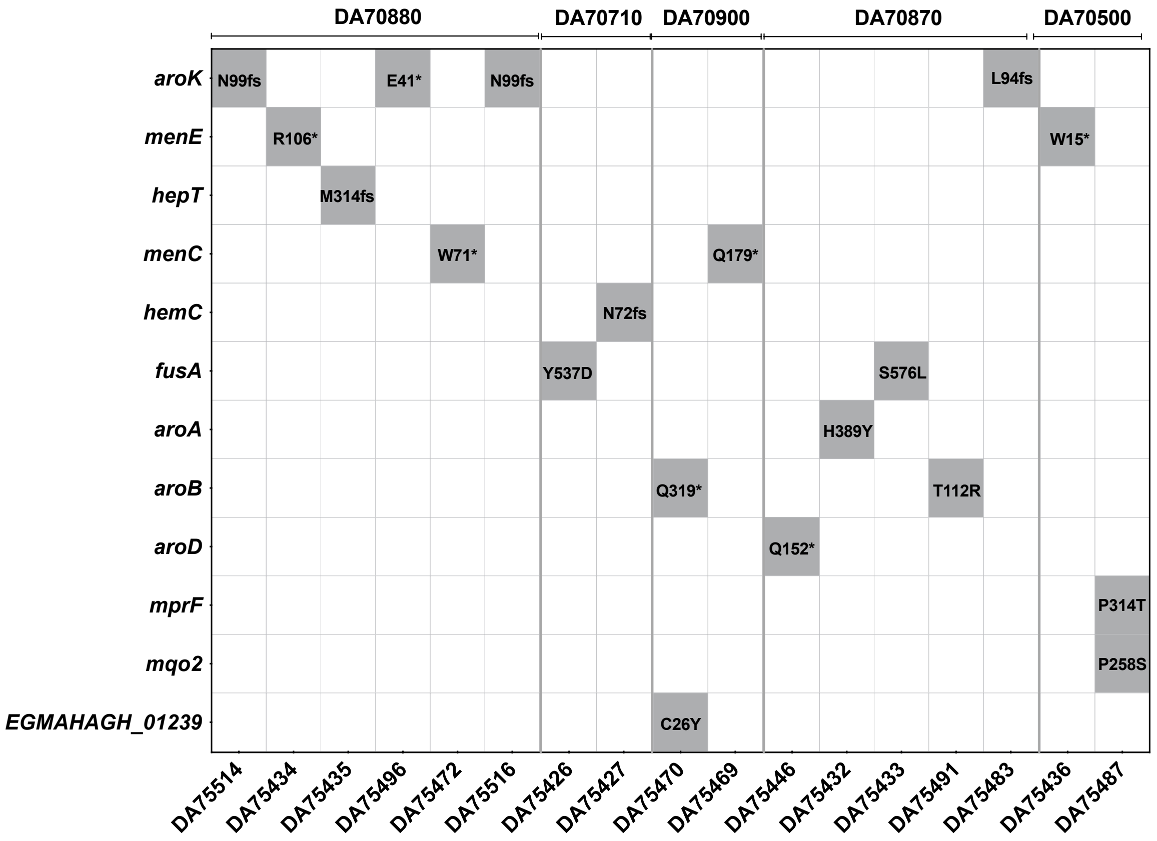

S9 Fig. **Mutations in GEN (gentamicin) resistant mutants.** Mutants ((DA number below) belong to parental isolates indicated on top of the graph. Fs indicates frame shift, and * stop-codon.
