# Supplementary material for: High prevalence of heteroresistance in Staphylococcus aureus is caused by a multitude of mutations in core genes
Source: PLoS Biol. 2024 Jan 4;22(1):e3002457. doi: 10.1371/journal.pbio.3002457 (PMC10766187; doi:10.1371/journal.pbio.3002457)
Supplement: S3 Table — (PDF) [file pbio.3002457.s014.pdf]

S3 Table. **Frequency of HR for each of the 40 clinical *S. aureus* isolates to six different antibiotics.** Gray cells represent HR phenotype determined by PAP test, and white cells correspond to non-HR phenotypes. Black cells are cases where the isolate was resistant to the antibiotic (MIC above the clinical breakpoint according to EUCAST). DAP (daptomycin), GEN (gentamicin), LNZ (linezolid), OXA (oxacillin), TEC (teicoplanin) and VAN (vancomycin).

Isolates DA number	DAP	GEN	LNZ	OXA	TEC	VAN	Number of HRs
DA70300							3
DA70302							1
DA70314							1
DA70318							1
DA70322							2
DA 70324							1
DA 70338							4
DA 70348							0
DA 70350							0
DA 70352							0
DA 70484							1
DA 70488							1
DA 70500							1
DA 70504							2
DA 70506							1
DA 70512							1
DA 70516							1
DA 70518							3
DA 70520							1
DA 70524							0
DA 70672							2
DA 70674							1
DA 70682							0
DA 70684							1
DA 70686							1
DA 70692							0
DA 70694							1
DA 70700							3
DA 70708							2
DA 70710							3
DA 70866							3
DA 70870							1
DA 70876							1
DA 70880							3
DA 70890							2
DA 70896							2
DA 70898							1
DA 70900							1
DA 70906							1
DA 70912							2
