# Supplementary material for: High prevalence of heteroresistance in Staphylococcus aureus is caused by a multitude of mutations in core genes
Source: PLoS Biol. 2024 Jan 4;22(1):e3002457. doi: 10.1371/journal.pbio.3002457 (PMC10766187; doi:10.1371/journal.pbio.3002457)
Supplement: S4 Table — (PDF) [file pbio.3002457.s015.pdf]

S4 Table. Antibiotic resistance genes in heteroresistant isolates used for selection and whole genome analysis of mutants. Heteroresistant isolates with resistance genes that potentially can lead to resistance towards the indicated antibiotics (none of the parental isolates are resistant to the indicated antibiotic from which the resistant mutants were selected). Resistance genes were searched using the comprehensive antibiotic resistance database (CARD). Dark gray cells represent the antibiotic for which selection of resistant mutant was carried out from the respective parental isolate. DAP (daptomycin), GEN (gentamicin), OXA (oxacillin), and TEC (teicoplanin).

Isolates DA number	DAP	GEN	OXA	TEC
DA70300		*ANT(9)-Ia*	*mgrA, blaZ*	
DA 70484			*mgrA, blaZ*	
DA 70500			*mgrA*	
DA 70504			*mgrA, blaZ*	
DA 70518			*mgrA, blaZ*	
DA 70520			*mgrA, blaZ*	
DA 70710			*mgrA*	
DA 70870		*APH (3')-IIIa*	*mgrA, blaZ*<br>*mecA, mecR*	
DA 70880			*mgrA*	
DA 70900			*mgrA, blaZ*	
