# Supplementary material for: High prevalence of heteroresistance in Staphylococcus aureus is caused by a multitude of mutations in core genes
Source: PLoS Biol. 2024 Jan 4;22(1):e3002457. doi: 10.1371/journal.pbio.3002457 (PMC10766187; doi:10.1371/journal.pbio.3002457)
Supplement: S11 Fig — Mutants (DA number below) belong to parental isolates indicated on top of the graph. Fs indicates frame shift, * stop-codon and Dup, duplication. (PDF) [file pbio.3002457.s011.pdf]

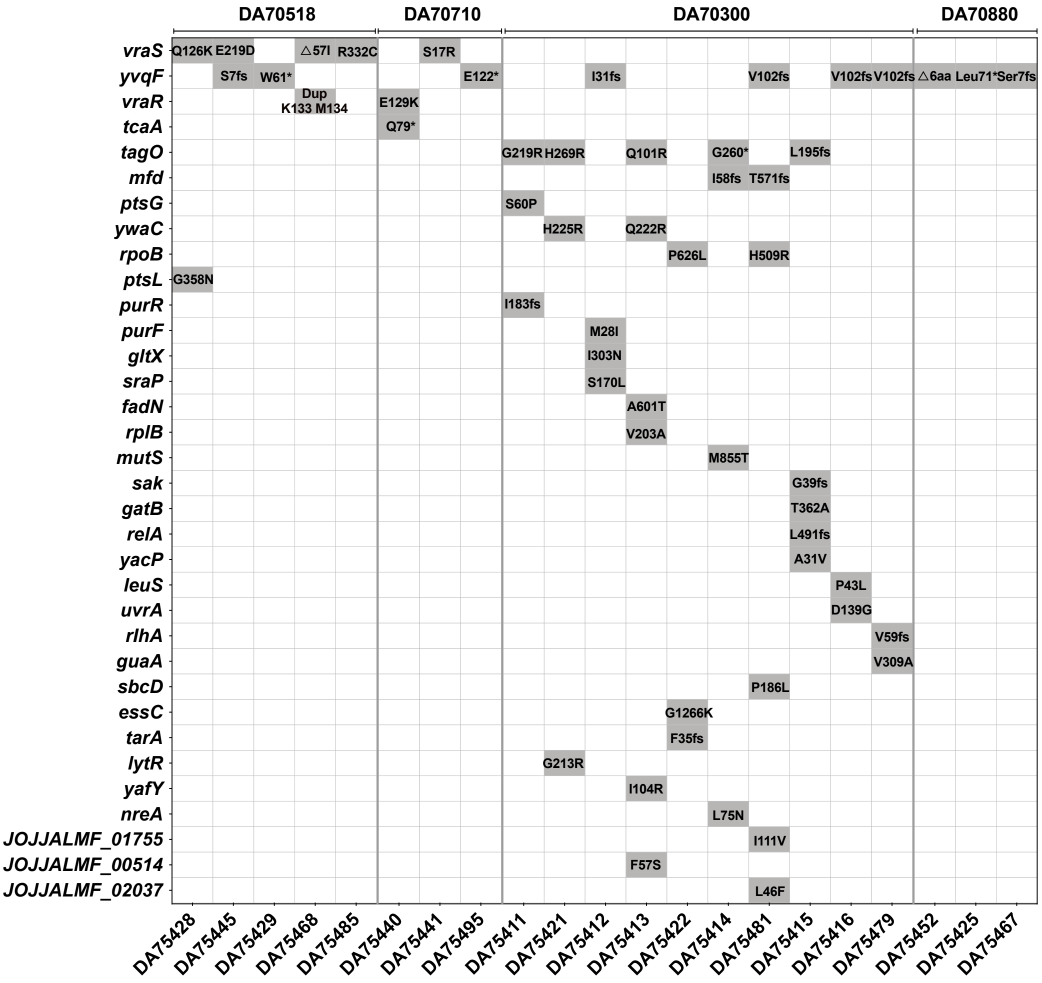

S11 Fig. Mutations in TEC (teicoplanin) resistant mutants. Mutants ((DA number below) belong to parental isolates indicated on top of the graph. Fs indicates frame shift, * stop-codon and Dup, duplication.
