# Supplementary material for: High prevalence of heteroresistance in Staphylococcus aureus is caused by a multitude of mutations in core genes
Source: PLoS Biol. 2024 Jan 4;22(1):e3002457. doi: 10.1371/journal.pbio.3002457 (PMC10766187; doi:10.1371/journal.pbio.3002457)
Supplement: Attachment — Submitted filename: Response to reviewers.pdf [file pbio.3002457.s016.pdf]

Response to reviewers

Reviewer #1: Protein evolution.

Reviewer #2: Antibiotic resistance.

Reviewer #1: This manuscript explores the mechanisms of heteroresistance, a relatively underexplored phenomenon in which a sensitive population consists of a small subpopulation of unstable resistant variants, across forty clinical Staphylococcus strains exposed to a variety of antibiotics. The authors reveal varying degrees of heteroresistance against gentamicin, oxacillin, daptomycin, and teicoplanin, with heteroresistance against gentamicin being particularly prevalent. The authors then go on to identify the mechanisms responsible for this heteroresistance by whole-genome sequencing selected resistant mutants from heteroresistant subpopulations, and found that mutations in different core chromosomal genes were responsible for generating these mutants. Finally, they investigate whether mutator phenotypes could be responsible for increased heteroresistance levels in Staphylococcus using fluctuation tests, but found no supporting evidence for this.

The topic of this study would be of interest to clinical microbiologists, particularly because information on heteroresistance in gram-positive bacteria is relatively limited. The manuscript is well-written, and the figures are appropriately employed. The methods and statistical analyses are clear and generally appropriate.

Comments
My main comment pertains to the definition of heteroresistance. I have consistently understood that a defining feature of heteroresistant variants is their high instability, with a tendency to revert back to susceptibility in the absence of selective pressure. This characteristic is also highlighted in the abstract and introduction of the manuscript. Therefore, I believe it is essential to demonstrate this instability in the putative heteroresistant variants. Although the authors have explored the fitness costs associated with these mutants and have found that some may exhibit significant fitness costs (which might facilitate rapid reversion), while others show little to no fitness cost, this alone doesn't establish the unstable nature of these variants.
If the authors argue that heteroresistant variants can also be stable, it becomes imperative to differentiate heteroresistant variants from regular resistant variants that originate from spontaneous mutations, either during the PAP analysis or more likely during the pre-culture. From my perspective, as an evolutionary biologist with a focus on resistance evolution, it seems that the authors have not identified true heteroresistant mutants but rather typical resistant mutants. Consequently, the variations in heteroresistance levels merely result from differences in mutation supply and/or target size for resistance to the specific antibiotic in question. In summary, the need to clearly distinguish between heteroresistance and regular resistance, particularly by demonstrating the instability of putative heteroresistant variants, is essential for a more accurate interpretation of the findings. If there is no difference, then I don't really understand the need for invoking heteroresistance.

The reviewer brings up a very important point that we in this field are discussing quite a bit and we fully understand the reviewer's puzzlement. One has to remember that the concept of heteroresistance (HR) is grounded in clinical microbiology and that it is a *phenotype* defined by two criteria: a frequency of the resistant sub-population >10^{-7} and a resistance level >8-fold of the susceptible population. As such its main use is to assess the potential negative impact HR might have on treatment (i.e. the higher the fraction of resistant cells and the higher their MIC the higher the risk for treatment failure). Used in this way the underlying mechanisms are not taken into account when determining if an isolate shows a HR phenotype or not (it is analogous to a MIC value, it is a phenotype, S or R, not a mechanism). Whether the resistant cells are unstable or not is not part of the definition, and it is sufficient for the two criteria mentioned above to be fulfilled to classify a strain as showing a HR phenotype (even though the underlying mechanism might just be regular resistance mutations).

With that said, recent work from us and others have shown that gene amplification (which is intrinsically unstable) is one of the main mechanisms for HR in Gram-negatives and instability has (for us and other researchers) become an additional criterium for defining this particular type of HR. However, it does not necessarily apply to all other mechanisms (e.g. regular resistance mutations). Thus, as the reviewer points out, for the latter instability is often observed if the fitness costs are high and compensatory mutations are available but the mutations can also be fully stable if there is a low/no fitness costs.

One could argue that the definition of HR should be updated and also include instability (to more clearly distinguish it from regular resistance mutations) but that is easier said than done since it would require clinical microbiology laboratories to introduce additional time-consuming and costly tests (which is unlikely to happen). It could also be argued that from a clinical perspective it is not that relevant whether the resistant sub-population is unstable or not since in both cases under selective pressure they might cause treatment failure.

To conclude, regular (stable or unstable) resistance mutations are also classified as showing a HR phenotype if they occur with a sufficiently high rate and provide sufficiently high resistance.

In the methods section discussing the PAP test, the authors mention that a 10-2 or 10-3 dilution was transferred to 1ml MHB (to generate a pre-culture from which samples would be taken for the PAP test). I would like to understand the reason behind this difference in dilution. It's worth noting that the additional generation in the 10-3 samples could potentially result in a higher number of variants, which might contribute to increased heteroresistance levels. Therefore, clarification on the rationale for this difference in dilution is warranted.
We apologize but this is a mistake from our side. It should be a 10E-3 dilution. This means that by inoculating 1ml of medium with 1ul of a 10E-3 dilution of an overnight culture (which contains about 10E9 cells per ml) gives an inoculum size of about 1000 cells. This is essential to assure that no pre-existing mutants are present when starting the test culture. Thus, all PAP cultures are inoculated in the same way. We have corrected this (lines 170-174).

Raw data from the PAP tests or whole-genome sequencing is not available.
This has been included.

Minor comments/ suggestions:
Fig S4: show the individual replicate data points or mean with error.
Done.

Fig S1: I would recommend transforming this scatterplot into a barplot for improved clarity. Additionally, adding the names of the antibiotics either within or above each corresponding panel would enhance the reader's experience, reducing the need to repeatedly refer to the legend (also for Fig 1, 2 and Fig S7 this would be helpful).
Done.

Reviewer #2: In this study, Heidarian and colleagues assess the potential for clinical isolates of Staphylococcus aureus to generate - in non-selective culture - a suite of mutants which are resistant to antibiotics. Both the frequency of these mutants in the population and the levels of resistance expressed by these mutants are high enough that many experimental populations can be identified as having "heteroresistance" (using standardized methods and categorizations). Resistant mutants were isolated, sequenced and the mutations conferring resistance were identified (but not directly tested for causality). Mutations underpinning the sub-population of resistant types were found across core genes. In gram-negative bacteria, such mutations might be found in genes which primarily degrade, alter or export the target antibiotic, or via amplification of such genes. The authors conclude that the mutational profile of mutations likely-causal of heteroresistance in these populations of S. aureus is probably due to 1) the lack of resistance genes in these isolates and 2) the lack of repetitive regions which would allow amplification of such genes.

I'm not very familiar with the HR literature, so I can't really comment as to the novelty or significance of the findings. The manuscript describes experiments with a suitable level of rigor, and the conclusions are reasonably supported with evidence. It is generally best to reconstruct mutations to prove functional causality, and the claims about the distinction of this gram positive and negative strain are made with reference to the literature, and are not directly tested. However, I'm not convinced such thoroughness is required for a brief report and I won't demand further experiments. Similarly, I'd like to see some points made more explicit or clarified (see below), but I won't demand extensive additions.

The reviewer is obviously correct that proper establishment of functional causality would require genetic reconstruction of each mutation. However, the repeated isolation of known (from literature) resistance mutations in independent selections we think provides a sufficiently strong link. In addition, reconstruction of mutations in clinical isolates of S. aureus can be technically challenging/impossible and it would require a very substantial work effort.

One thing that does strongly need attention is a lack of data availability (see point 1 below). PLOS does have a clear data policy "All data and related metadata underlying reported findings should be deposited in appropriate public data repositories, unless already

provided as part of a submitted article". There is no raw genomic data provided in the supplementary files, which should really be provided. Reproducibility of most of the findings is contingent on having exactly these strains and it's a bit of a push to expect they are shared considering they are pathogenic. Regardless, the authors should at least provide the raw sequencing data so claims about the lack of resistance genes/repetitive regions in these strains can be independently assessed.
Raw sequencing data has been uploaded (line 228) and raw data for PAP curves has been added (new supplementary Table 1).

Subsequent points (2-7) describe areas that require attention to avoid confusion from a reader's perspective.

1. It's claimed in the author statements that all raw data are available in the supplementary files, however, considerable amounts of raw genomic data are obviously not present. I assume that the raw dataset (raw genomic sequencing files) will be uploaded to a repository/genbank upon publication of this MS. This is important, especially so readers could assess the claims about the lack of repetitive regions in S. aureus. Very helpful files to include in this dataset would be the reference sequence made of the parental isolates.
We apologize for this oversight. Raw sequencing data of all parental and mutant strains has now been uploaded (and accession numbers added) and raw data for the PAP curves has been added (new supp Table S1).

2. There are several references to "stability" in the abstract and introduction (paragraphs 1 and 3). This theme of stability is not explicitly returned to in the discussion. I assume the HR-causing mutations described for these population of S. aureus are stable (and require secondary mutation to allow compensation of their deleterious effects in the absence of selection). Please explicitly discuss the "stability" of the mutations seen here (i.e. the likelihood that the exact mutation reverts to WT) and what this means for the subsequent evolution of S. aureus (i.e. are compensatory mutations expected). Alternatively, remove the stability concept from the introduction and abstract.
We introduced instability in the Introduction as background information to specifically indicate that gene amplification mediated heteroresistance (HR) is highly unstable but that mutation-mediated can be either unstable (if the fitness costs are high and compensatory mutations are available) but they might also be fully stable if there is a low/no fitness costs. We have added a comment regarding the stability of the resistant mutants found in this study (lines 392-394).

3. I was confused on the first reading with the various references to "Isolates". The results start with a description of "isolates", and I misinterpreted this to mean an analysis of HR isolates (not parental clonal isolates). Please more strongly distinguish parental isolates and HR isolates.
Good point. We have added clarifications at several places to distinguish clearly between parental isolates and mutants obtained from these isolates (lines 270,279-281, 313, Table 2 and legend).

4. Similarly, it is helpful for the reader to realize (early in the results) how the HR types were generated without reference to the methods. For those unfamiliar with HR, it was not

obvious in what conditions these HR types evolved. The diversity of HR types is generated from a limited population size with relatively few generations (<20 generations) in the absence of selection. I suggest it is made clearer that the HR types evolved under such conditions. This would allow a better understanding of the conduct of the experiments, and makes the high diversity of HR types even more surprising.
We have added a clarification in Mat-Met (lines 170-174).

5. There is repeated reporting of the "prevalence frequencies" (in the abstract, introduction, results section "Prevalence of HR varies depending on antibiotic", the Y-axis of Fig S3 etc). The prevalence of the isolates is potentially misleading. Reported is the prevalence of cultures within which HR types are identified. It could be interpreted that the prevalence is the proportion of HR types within populations (as shown in Figure 1, Y-xis.). Please be more explicit that the "prevalence frequency" means the "frequency of cultures with HR".
We have clarified this (lines 279-281, Table 2 legend). The prevalence is the fraction in % of the 40 different parental isolates examined showed a HR phenotype as determined by PAP tests.

6. While there is a very detailed description of the predicted function of each mutant locus and how this may provide resistance to each AB, lacking is a more general discussion of the classes of mutations selected by the antibiotics. It appears that loss-of-function mutations are generally more prevalent, and as partially suggested in the discussion, it seems LNZ and VAN cannot be caused by such mutations. A more general interpretation of the data is a that HR is made possible if large numbers of resistant mutants are generated before plating on selective antibiotics (ie, the large target size of these target genes aids the production of HR types). I'd suggest introducing this framing in your description of these mutations as it might provide a broader appeal to those interested in genetics.
Good point. We have added a short section regarding this (lines 386-394).

7. Section "Differences between S. aureus and Gram-negative bacteria in resistance mechanisms". The authors hypothesize that the presence of resistance of resistance genes alters the mechanism by which HR occurs in gram-negative bacteria. However, there's no reason to believe mutations to core genes (as described here) is not occurring in gram-negative species, but they are rarely selected because "better" solutions (amplification of resistance genes) are found. It would be an idea (which the authors do not need to implement) to directly state this, which allows the testable predication that HR-mutants are to be expected in gram negative bacteria which lack resistance genes.
Yes, the reviewer is correct that in Gram-negatives both mechanisms occur (amplification and mutations in core genes, but simply because of the much higher rates of amplification they are mainly observed (see Nicoloff, Hjort and Andersson Nature Microbiology 2019).

Excellent point about testing this but it might be hard to find such Gram-negatives. Another potential way to test this is to delete resistance genes from a Gram-negative bacterium that shows HR and then determine if gene amplifications are absent and instead regular point mutations are dominantly observed (and probably loss of the HR phenotype).
